# Early-onset colorectal cancer burden in China from 1990 to 2023: a systematic analysis based on the Global Burden of Disease Study 2023

**DOI:** 10.3389/fonc.2026.1958227

**Published:** 2026-10-12

**Authors:** Haijian Wei, Hao Wu, Shuai Wang, Yiwei Yao, Kaiwen Zheng, Zhini Yang, Jianan Ma

**Affiliations:** 1 Department of Organ Transplantation, The Affiliated Yantai Yuhuangding Hospital of Qingdao University, Yantai, China; 2 Key Laboratory of Organ Regeneration and Transplantation of the Ministry of Education, The First Hospital of Jilin University, Changchun, China; 3 Department of Urology, The First Hospital of Jilin University, Changchun, China; 4 Department of Gastric and Colorectal Surgery, General Surgery Center, The First Hospital of Jilin University, Changchun, China; 5 Cancer Center, The First Hospital of Jilin University, Changchun, China; 6 Anaesthesiology, Medical College of Yanbian University, Yanji, China; 7 Department of Pathology, School of Basic Medical Sciences, Shandong Medical and Pharmaceutical University, Yantai, China

**Keywords:** China, disease burden, early-onset colorectal cancer, forecasting, global burden of disease, risk attribution, temporal trend

## Abstract

**Background:**

Early-onset colorectal cancer (EOCRC) impairs the health of China’s working-age population and causes substantial socioeconomic costs. National long-term epidemiological data integrating trend segmentation, risk attribution and medium-term forecasting for domestic EOCRC remain insufficient. This study analysed China’s 33-year EOCRC burden (1990-2023), identified trend inflection points, quantified modifiable risk-attributable burden and predicted epidemic trends to support tiered prevention strategies.

**Methods:**

Data on EOCRC burden among Chinese residents aged 15–49 were derived from the Global Burden of Diseases, Injuries, and Risk Factors Study 2023. We analyzed absolute cases and age-standardized rates of incidence, mortality, DALYs and prevalence. Joinpoint regression detected segmented nonlinear trends, and Autoregressive integrated moving average (ARIMA) models projected burden for 2024-2040. Dietary, metabolic and behavioural risk-attributable burden was quantified.

**Results:**

From 1990 to 2023, national EOCRC mortality and DALY burden decreased continuously, whereas incident and prevalent cases increased significantly. Gender and age gradients existed: males and residents aged 45–49 carried the highest disease burden with slower improvement. Low milk and whole grain intake were the leading risk contributors, while high processed meat and high BMI showed rapidly rising attributable burden. All four burden indicators presented multi-stage trends and rebounded after 2020. ARIMA forecasts indicated age-standardized mortality and DALY rates would keep decreasing by 2040, yet male-dominated burden gaps remain prominent.

**Conclusion:**

China’s EOCRC presents divergent long-term burden trends and stable age-sex disparities. The high burden in the 45–49 age group supports prioritizing this population for further screening evaluation, and male-oriented lifestyle interventions may help curb growing incidence and alleviate youth colorectal cancer burden.

## 1 Introduction

Colorectal cancer (CRC) ranks among the most prevalent malignant neoplasms worldwide and constitutes a top leading cause of cancer-related mortality, imposing enormous public health burdens and socioeconomic losses across global populations (1). China bears a substantial share of the global CRC burden, accounting for over one-third of new CRC cases and cancer deaths each year (2). While overall CRC epidemiological indicators in elderly populations have gradually improved over the past decades, early-onset colorectal cancer (EOCRC), uniformly defined as CRC diagnosed in individuals aged 15–49 years, has attracted growing scholarly attention due to its unique clinical manifestations and divergent epidemiological characteristics (3, 4). Compared with late-onset colorectal cancer, EOCRC is characterized by more aggressive pathological phenotypes, advanced tumor stages at initial diagnosis, and inferior long-term survival outcomes (5). Moreover, EOCRC predominantly affects working-age young and middle-aged groups, leading to massive premature labor loss and heavy economic pressure on individual families, making it an urgent public health priority requiring targeted prevention and intervention strategies (6, 7).

Cumulative population-based epidemiological evidence has demonstrated prominent heterogeneity in EOCRC incidence and mortality trends across regions, ethnic groups, and sex subgroups (8). Obvious sex disparities exist in EOCRC disease burden within China: males experience rapidly rising incidence and prevalence, while females present sustained declines in mortality, disability-adjusted life years (DALYs) and incidence metrics, resulting in a continuously widening gender gap in disease burden (9, 10). Multiple modifiable dietary, metabolic and behavioral exposures are confirmed independent risk contributors to EOCRC, including insufficient intake of milk, whole grains and calcium, excessive consumption of processed and red meat, high body-mass index (BMI), smoking, and heavy alcohol use; these divergent lifestyle exposures partially explain the observed sex-specific disparities in EOCRC burden (11, 12). In addition, distinct age-gradient patterns of EOCRC burden have been widely documented, with disease risk rising steadily with age and peaking in the 45-49-year subgroup, whereas adolescents and young adults aged 15–34 carry relatively mild disease burden (13). Nevertheless, most existing domestic studies mainly focus on overall CRC or single-center hospital clinical cohorts, yet few nationwide long-term systematic analyses simultaneously combine temporal trend description, demographic disparity assessment, quantitative risk burden estimation, staged inflection point screening and long-range burden forecasting targeted at Chinese EOCRC populations (14, 15).

The Global Burden of Diseases, Injuries, and Risk Factors Study (GBD) provides standardized, multi-year population-level estimates of CRC incidence, prevalence, mortality, and DALYs for all countries worldwide, offering reliable, consistent data sources for long-term national epidemiological trend analysis (16). Although numerous GBD-based studies have explored the overall digestive tract cancer burden in China, few have specifically focused on comprehensive multi-dimensional epidemiological assessments of EOCRC. Several recent GBD-based studies have nonetheless described the burden and projected trends of EOCRC in China (9, 10, 12, 13), yet these works have notable limitations. Huang et al. (9), Shao et al. (10), and Ong et al. (13) focused on China but used earlier GBD versions (GBD 2019 or 2021) and addressed only selected dimensions of the EOCRC burden, whereas Jin et al. (12) adopted the latest GBD 2023 data but framed China as one of five East Asian populations in a cross-national comparison, with limited depth in China-specific five-year age stratification, multi-risk-factor attribution, and segmented inflection-point analysis. Building on the GBD 2023 dataset (1990-2023), the present study extends this prior work in several important respects. First, it delivers a China-specific, full-spectrum assessment that integrates the above analytical dimensions within a single framework, rather than addressing them separately or partially as in prior studies. Second, it reports joinpoint-derived multi-stage trends and statistically tested inflection points-including the post-2020 rebound-that were not systematically provided in earlier national EOCRC analyses. Third, it quantifies China-specific age-standardized attributable mortality and DALY rates for multiple modifiable dietary, metabolic and behavioural exposures, complementing comparative or diet-only analyses in earlier work.

Therefore, based on the latest GBD 2023 global dataset, this study conducted a comprehensive epidemiological analysis of EOCRC burden among Chinese residents aged 15–49 years from 1990 to 2023. We aimed to quantify the long-term changing trajectories of national absolute disease burden and age-standardized rates (ASRs); systematically explore epidemiological disparities across sex and five-year age subgroups; evaluate the attributable burden magnitude and age distribution patterns of multiple modifiable dietary, metabolic and behavioral risk factors; identify nonlinear phased temporal trends and critical inflection points via Joinpoint regression analysis; and further predict EOCRC mortality and DALY burden trends up to 2040 using the ARIMA model. This study intends to systematically elaborate the full-spectrum epidemiological characteristics of EOCRC in China over the past 33 years, provide evidence-based references for optimizing youth-targeted cancer prevention, high-risk population stratified screening, and rational healthcare resource allocation, and support the development of precise EOCRC control policies to mitigate future EOCRC disease burden.

## 2 Methods

### 2.1 Data source and acquisition

Data for this study were extracted from the GBD 2023 official database via the GBD Results Tool on the VizHub platform. The GBD research system integrates multi-source epidemiological raw data, covering national tumor registry databases, population mortality surveillance records, large-scale population cohort evidence, and hospital-based epidemiological statistics. A unified standardized global modelling framework is adopted to generate harmonized, comparable burden estimates across different regions, populations and long time series (17). All GBD-derived burden estimates were extracted with their corresponding 95% uncertainty intervals (UIs).

This study targeted EOCRC in Chinese residents aged 15–49 years, with the research time window spanning 1990 to 2023. Four core epidemiological outcome indicators were extracted for subsequent quantitative analysis: incident cases stood for newly diagnosed CRC cases within a single calendar year; mortality cases referred to deaths directly attributed to CRC; DALYs were calculated as the summation of years of life lost (YLLs) from premature cancer death and years lived with disability (YLDs) caused by CRC-related lesions and clinical symptoms (18); prevalent cases represented the cumulative number of surviving patients diagnosed with CRC at a specific time point.

### 2.2 Study population and grouping settings

Consistent with the mainstream epidemiological definition of EOCRC, individuals aged 15–49 years were defined as the target research population in this study (19). Stratified analyses were performed to explore heterogeneous epidemiological characteristics across diverse population subgroups. First, the total population was divided into male and female subgroups to quantify gender disparities in the overall burden of EOCRC. For age stratification, the whole population was split into seven successive 5-year age brackets (15-19, 20-24, 25-29, 30-34, 35-39, 40-44, 45–49 years old), so as to fully depict the gradient variation law of EOCRC burden with age growth.

Furthermore, this study quantified the attributable burden of multiple recognized modifiable dietary, metabolic and behavioral risk factors closely correlated with EOCRC progression, including insufficient intake of milk, whole grains and dietary fiber, inadequate calcium intake, excessive consumption of red meat and processed meat, elevated BMI, cigarette smoking and heavy alcohol drinking (20). The temporal trends and age distribution features of the burden attributable to the above risk factors were further compared and analyzed.

### 2.3 Definition of core indicators

All ASRs in the present study were standardized against the GBD 2019 global standard population. This standardization approach can effectively eliminate the confounding effect brought by chronological changes in population age composition and guarantee the longitudinal comparability of long-term epidemiological trend data (16). The standardized indicators involved in this study included age-standardized incidence rate (ASIR), age-standardized mortality rate (ASMR), age-standardized DALY rate (ASDR) and age-standardized prevalence rate (ASPR), and all rate indicators were uniformly expressed per 100,000 persons. The percentage change of absolute burden metrics between 1990 and 2023 was calculated with the following formula: Percentage change = [(Value_2023_ − Value_1990_)/Value_1990_] × 100%.

### 2.4 Statistical analysis

#### 2.4.1 Joinpoint regression trend analysis

Joinpoint segmented log-linear regression model was adopted to fit the long-term dynamic trends of all age-standardized burden indicators from 1990 to 2023. This statistical method can accurately screen statistically significant trend inflection points and divide the whole research period into multiple independent segments with distinct rising or declining speeds (21, 22).

Joinpoint analyses were performed using the Joinpoint Regression Program (Version 5.2.0.0; National Cancer Institute, Calverton, MD, USA). The maximum number of joinpoints was set to three, and model selection was conducted using the Monte Carlo permutation test at a significance level of 0.05 (21). A log-linear model was fitted by taking the natural logarithm of each age-standardized burden indicator, with the calendar year as the independent variable. For each identified segment, the annual percentage change (APC) was calculated as APC = 100 × (exp(β) - 1), where β is the slope of the corresponding segment. To summarize the overall trend over the entire study period, the average annual percentage change (AAPC) was computed as a weighted average of segment-specific APCs, using the number of years in each segment as weights: AAPC = 100 × (exp(Σ(w_i_ × β_i_)/Σw_i_) - 1), where w_i_ and β_i_ denote the number of years and the slope of the i-th segment, respectively (23). Combined with the corresponding 95% confidence intervals (CIs), the trend property of each indicator was judged: the trend was defined as statistically increasing if the entire 95% CI was above zero; statistically decreasing if the whole 95% CI was below zero; and stable if the 95% CI contained zero (24–26).

#### 2.4.2 Risk factor attributable burden analysis

On the basis of the comparative risk assessment framework embedded in GBD 2023, this study quantitatively evaluated EOCRC deaths and DALYs attributable to the above dietary, metabolic and behavioral risk factors, as well as matched age-standardized attributable burden rates (27). On this basis, the temporal evolution characteristics and age distribution differences of attributable burden among different risk factors were further compared and summarized. Notably, the current GBD comparative risk assessment only incorporates modifiable dietary, metabolic, and behavioral exposures. Non-modifiable risk contributors, including genetic susceptibility, family cancer history, and inflammatory bowel disease, are not captured by this framework, and thus their attributable burden was not assessed in the present study. In addition, all attributable burden estimates represent population-level statistical associations rather than individual-level causal effects.

#### 2.4.3 ARIMA time series prediction

ARIMA modelling was performed using the forecast package in R (Version 4.5.1). Stationarity of each time series was assessed using the Kwiatkowski-Phillips-Schmidt-Shin (KPSS) test (28), with non-stationary series differenced as needed. Candidate ARIMA (p,d,q) models-where p, d, and q denote the orders of autoregression, differencing, and moving average, respectively-were identified via auto.arima() (stepwise = FALSE, approximation = FALSE) (29) and selected by the lowest Akaike Information Criterion (AIC). Model parameters were estimated by maximum likelihood, and residual adequacy was verified using the Ljung-Box test (30) with visual inspection of residual autocorrelation function (ACF) and quantile-quantile (Q-Q) plots. The optimal models were fitted for ASRs and absolute numbers of mortality and DALYs by sex, and all model specifications and diagnostic results are summarized in **Supplementary Table 1**. Out-of-sample validation was performed by fitting models on 1990–2018 data and forecasting 2019-2023, with accuracy assessed by root mean square error (RMSE), mean absolute error (MAE), and mean absolute percentage error (MAPE). All models demonstrated reasonable forecasting accuracy, with MAPE ranging from 3.1% to 6.7% (**Supplementary Table 2**) (31, 32). The optimal models, fitted on the full 1990–2023 dataset, were used to project 2024–2040 burden with 95% prediction intervals (PIs).

It should be noted that ARIMA forecasting extrapolates historical trends on the assumption that risk-factor profiles, screening policies and therapeutic performance will not undergo major shifts over the projection period. In addition, ARIMA forecasting was restricted to mortality and DALY metrics in the present study. Preliminary modelling attempts for incidence and prevalence were unsuccessful due to data-driven convergence errors and unsatisfactory model performance; reliable long-term projections for these two outcomes could not be generated.

#### 2.4.4 Software application

Basic data collation, descriptive statistical calculation and ARIMA model prediction analysis were accomplished by R software (Version 4.5.1). Joinpoint segmented trend fitting and AAPC value calculation were finished using Joinpoint Regression Program (Version 5.2.0.0). All statistical graphs and trend charts in this research were drawn relying on the built-in visualization packages of R software.

### 2.5 Ethical statement

All data adopted in this research are public population-level aggregated disease burden data officially released by the GBD platform. This study did not collect individual clinical specimen data or implement human-related intervention experiments; hence, formal ethical approval and individual written informed consent were not required. All data analysis procedures of this research were carried out in strict accordance with the basic principles stated in the Declaration of Helsinki.

## 3 Results

### 3.1 Overall burden of EOCRC in China

From 1990 to 2023, EOCRC burden among Chinese individuals aged 15–49 years exhibited divergent trends across core metrics (**Table 1**). Deaths and DALYs fell by 26.34% and 31.40% nationwide, with significant declining AAPCs for ASMR (-0.86) and ASDR (-1.03). In contrast, incident and prevalent cases rose by 18.60% and 55.67%, accompanied by significant upward AAPCs of ASIR (0.57) and ASPR (1.40). Overall, mortality and premature loss burden decreased over the 33 years, yet the continuous growth of national incident and prevalent cases has led to the expanding cumulative disease burden of EOCRC in China.

**Table 1:** Age-standardized rates and burden of EOCRC in China, 1990-2023.

Indicator	Sex	Number (1990, 95%UI)	Number (2023, 95%UI)	Percent change (%)	ASR (1990, 95%UI)	ASR (2023, 95%UI)	AAPC of ASR (95% CI)
Death	Both	26532(21438,31384)	19544(16199,23570)	-26.34	3.97(3.21,4.69)	3.03(2.51,3.65)	-0.86 (-1.10 to -0.61)*
	Female	11177(7618,13851)	5470(4265,7462)	-51.06	3.46(2.36,4.29)	1.77(1.38,2.42)	-2.02 (-2.24 to -1.79)*
	Male	15355(11398,19846)	14074(10993,17669)	-8.34	4.44(3.30,5.74)	4.17(3.26,5.24)	-0.10 (-0.33 to 0.13)
DALY	Both	1412580(1143834,1685144)	968966(811648,1165778)	-31.40	211.26(171.06,252.02)	150.08(125.71,180.56)	-1.03 (-1.27 to -0.79)*
	Female	599067(405507,745157)	271883(214175,366161)	-54.62	185.43(125.52,230.65)	88.12(69.42,118.68)	-2.23 (-2.46 to -2.00)*
	Male	813513(595538,1056113)	697083(550030,866741)	-14.31	235.40(172.33,305.60)	206.78(163.16,257.11)	-0.28 (-0.51 to -0.05)*
Incidence	Both	40595(32950,46663)	48144(38339,60700)	18.60	6.07(4.93,6.98)	7.46(5.94,9.40)	0.57 (0.41 to 0.73)*
	Female	16744(11270,21008)	12410(9192,16789)	-25.88	5.18(3.49,6.50)	4.02(2.98,5.44)	-0.89 (-1.11 to -0.67)*
	Male	23852(17694,30696)	35734(26643,46355)	49.82	6.90(5.12,8.88)	10.60(7.90,13.75)	1.25 (1.03 to 1.47)*
Prevalence	Both	188933(148339,223093)	294121(229210,353678)	55.67	28.26(22.18,33.36)	45.56(35.50,54.78)	1.40 (1.27 to 1.52)*
	Female	75014(50958,94506)	74063(56318,100031)	-1.27	23.22(15.77,29.25)	24.01(18.25,32.42)	0.03 (-0.15 to 0.20)
	Male	113919(84871,146182)	220058(167703,273124)	93.17	32.96(24.56,42.30)	65.28(49.75,81.02)	2.12 (1.95 to 2.28)*

AAPC, average annual percentage change; ASR, age-standardized rate; CI, confidence interval; DALY, disability-adjusted life year; UI, uncertainty interval. *P < 0.05.

### 3.2 Sex disparities in EOCRC burden

Males and females presented strikingly divergent opposite trends in EOCRC burden from 1990 to 2023, with prominent sex gaps in absolute counts and ASRs across all four indicators (**Table 1**, **Figures 1A–D**). For mortality and DALY metrics, both sexes saw declining absolute burden, yet females experienced far larger reductions than males: female deaths dropped by 51.06% compared with an 8.34% decline in males, while female DALYs decreased by 54.62%, versus a mild 14.31% reduction among males. Consistently, the annual declines of ASMR and ASDR were much more pronounced in females, whereas male ASMR showed no statistically significant long-term downward trend (**Table 1**, **Figures 1A, B**). Incidence and prevalence burden showed completely opposite sex-specific trajectories (**Table 1**, **Figures 1C, D**): male incident and prevalent cases surged by 49.82% and 93.17%, accompanied by significant rising ASIR and ASPR, while female incident cases fell by 25.88% with a sustained drop in ASIR, and female prevalence remained nearly unchanged with a non-significant flat ASPR trend. The long-term sex-stratified temporal variations of absolute case numbers and standardized rates are visually displayed in **Figures 1A–D**.

**Figure 1:**
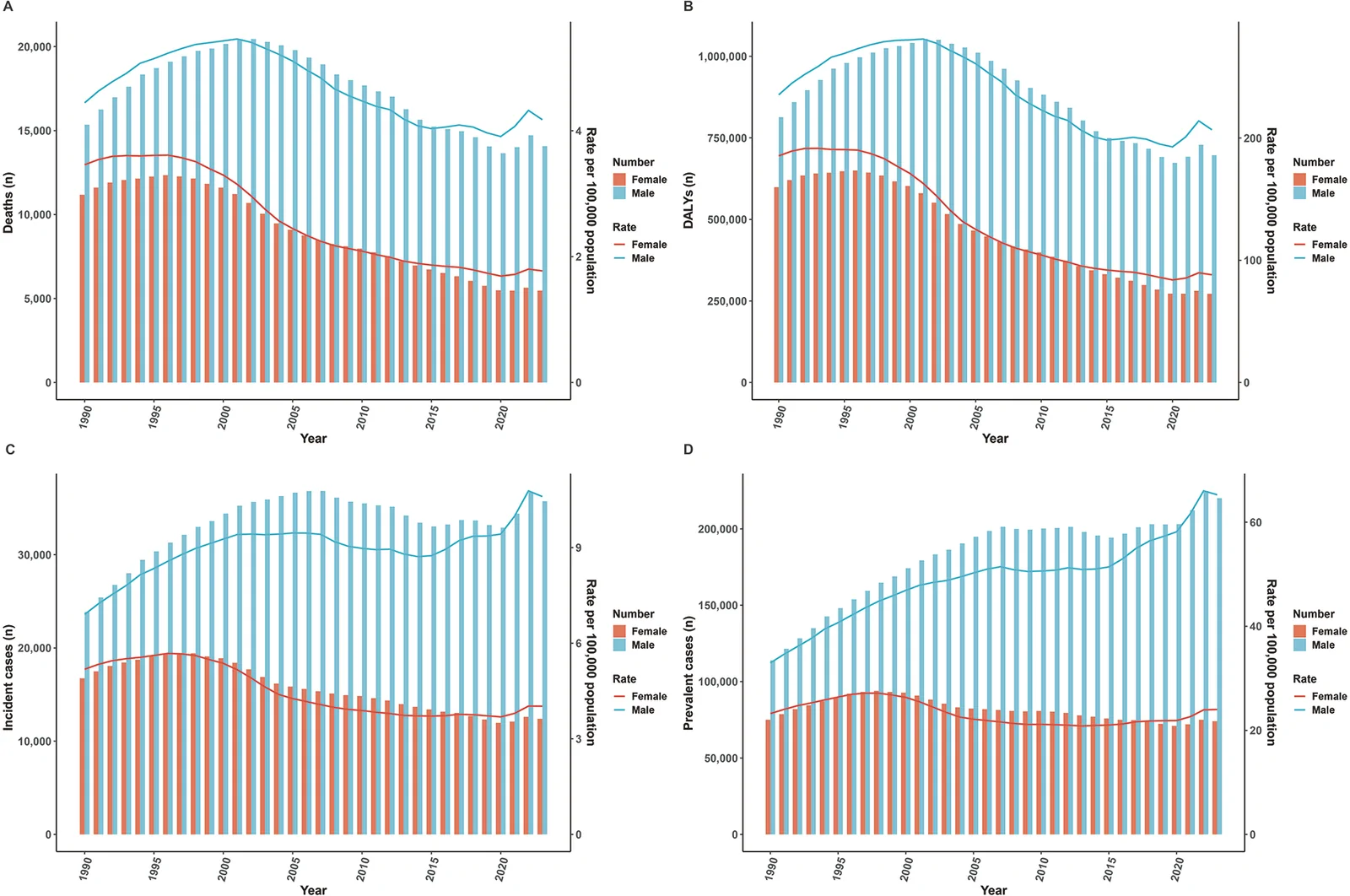
Temporal trends in the burden of EOCRC in China among individuals aged 15–49 years, stratified by sex (1990-2023). Panels show **(A)** deaths, **(B)** DALYs, **(C)** incident cases, and **(D)** prevalent cases. Bars represent the absolute number of each measure, while lines represent the corresponding ASRs per 100,000 population. Blue bars and lines denote males, and red bars and lines denote females, as shown in the legend. DALY, disability-adjusted life year.

### 3.3 Age-specific trends in EOCRC burden

From 1990 to 2023, age-specific EOCRC burden among Chinese people aged 15–49 years varied markedly across seven 5-year age brackets (**Table 2**). Cross-sectional data in 1990 and 2023 demonstrated a clear age gradient for all four burden metrics, with absolute case numbers and standardized rates rising steadily with age (**Supplementary Figures S1A–D**, **Figures 2A–D**).

**Table 2:** Age-specific numbers and rates of EOCRC burden in China, 1990-2023.

Metric	Measure	Number (1990, 95%UI)	Number (2023, 95%UI)	Percent change (%)	ASR (1990, 95%UI)	ASR (2023, 95%UI)	AAPC of ASR (95% CI)
Death	15–19 years	1081(779,1348)	215(156,280)	-80.07	0.85(0.61,1.06)	0.27(0.19,0.35)	-3.11 (-3.49 to -2.73)*
	20–24 years	1804(1318,2340)	258(192,339)	-85.67	1.36(1.00,1.77)	0.37(0.27,0.48)	-3.68 (-4.08 to -3.28)*
	25-29years	2388(1750,2960)	643(489,847)	-73.05	2.17(1.59,2.69)	0.82(0.63,1.09)	-2.74 (-2.97 to -2.50)*
	30-34years	3415(2622,4214)	1724(1334,2226)	-49.51	3.86(2.96,4.76)	1.61(1.24,2.07)	-2.50 (-2.76 to -2.25)*
	35–39 years	5256(3928,6547)	3296(2576,4339)	-37.29	5.74(4.29,7.15)	2.79(2.18,3.67)	-1.93 (-2.29 to -1.57)*
	40-44years	5544(4312,6741)	4448(3465,5780)	-19.77	8.24(6.41,10.02)	4.89(3.81,6.36)	-1.56 (-1.75 to -1.36)*
	45-49years	7045(5806,8364)	8959(6922,11345)	27.17	13.61(11.22,16.16)	8.98(6.94,11.37)	-1.25 (-1.49 to -1.00)*
DALY	15–19 years	79385(57206,98715)	16211(11926,20994)	-79.58	62.51(45.04,77.73)	20.12(14.80,26.06)	-3.04 (-3.42 to -2.66)*
	20–24 years	123228(90062,159828)	17881(13311,23371)	-85.49	93.09(68.04,120.74)	25.27(18.81,33.03)	-3.49 (-3.89 to -3.10)*
	25-29years	151822(111220,187910)	41000(31114,53749)	-72.99	137.80(100.95,170.56)	52.57(39.90,68.92)	-2.73 (-2.96 to -2.51)*
	30-34years	199278(152934,245149)	101406(78245,129999)	-49.11	225.17(172.81,277.00)	94.40(72.84,121.02)	-2.48 (-2.75 to -2.21)*
	35–39 years	281853(210713,351774)	178435(140408,234578)	-36.69	307.82(230.13,384.18)	150.94(118.77,198.43)	-1.91 (-2.27 to -1.56)*
	40-44years	269455(209662,327371)	218350(170902,282301)	-18.97	400.45(311.59,486.52)	240.09(187.92,310.41)	-1.54 (-1.74 to -1.34)*
	45-49years	307559(252723,365332)	395683(308622,498315)	28.65	594.24(488.29,705.86)	396.69(309.41,499.59)	-1.07 (-1.31 to -0.83)*
Incidence	15–19 years	1970(1435,2420)	821(544,1095)	-58.31	1.55(1.13,1.91)	1.02(0.68,1.36)	-1.16 (-1.49 to -0.83)*
	20–24 years	2619(1867,3394)	640(441,856)	-75.55	1.98(1.41,2.56)	0.91(0.62,1.21)	-2.10 (-2.46 to -1.73)*
	25-29years	3558(2641,4412)	1332(999,1793)	-62.56	3.23(2.40,4.00)	1.71(1.28,2.30)	-1.81 (-2.03 to -1.59)*
	30-34years	5506(4178,6779)	3986(3050,5270)	-27.60	6.22(4.72,7.66)	3.71(2.84,4.91)	-1.46 (-1.69 to -1.22)*
	35–39 years	8138(6084,10138)	7952(5974,10790)	-2.28	8.89(6.64,11.07)	6.73(5.05,9.13)	-0.60 (-0.95 to -0.26)*
	40-44years	8248(6463,10070)	10791(8179,14428)	30.84	12.26(9.61,14.97)	11.87(8.99,15.86)	-0.02 (-0.27 to 0.22)
	45-49years	10555(8630,12488)	22620(16703,29134)	114.30	20.39(16.67,24.13)	22.68(16.75,29.21)	0.39 (0.12 to 0.66)*
Prevalence	15–19 years	9985(6669,12974)	5693(4017,8196)	-42.99	7.86(5.25,10.22)	7.07(4.99,10.17)	-0.54 (-0.87 to -0.21)*
	20–24 years	10968(7686,14097)	4124(2994,5654)	-62.40	8.29(5.81,10.65)	5.83(4.23,7.99)	-0.85 (-1.18 to -0.52)*
	25-29years	15689(11404,19489)	7759(5797,10107)	-50.54	14.24(10.35,17.69)	9.95(7.43,12.96)	-1.04 (-1.25 to -0.84)*
	30-34years	26406(19994,31910)	24262(18251,31916)	-8.12	29.84(22.59,36.06)	22.59(16.99,29.71)	-0.72 (-0.97 to -0.47)*
	35–39 years	38500(30105,47191)	48624(37196,63771)	26.30	42.05(32.88,51.54)	41.13(31.46,53.94)	0.01 (-0.21 to 0.24)
	40-44years	38220(30562,46212)	65646(49097,82639)	71.76	56.80(45.42,68.68)	72.18(53.98,90.87)	0.76 (0.57 to 0.96)*
	45-49years	49165(38771,58567)	138014(102625,171847)	180.72	94.99(74.91,113.16)	138.37(102.89,172.29)	1.21 (1.00 to 1.42)*

AAPC, average annual percentage change; CI, confidence interval; UI, uncertainty interval. *P < 0.05.

**Figure 2:**
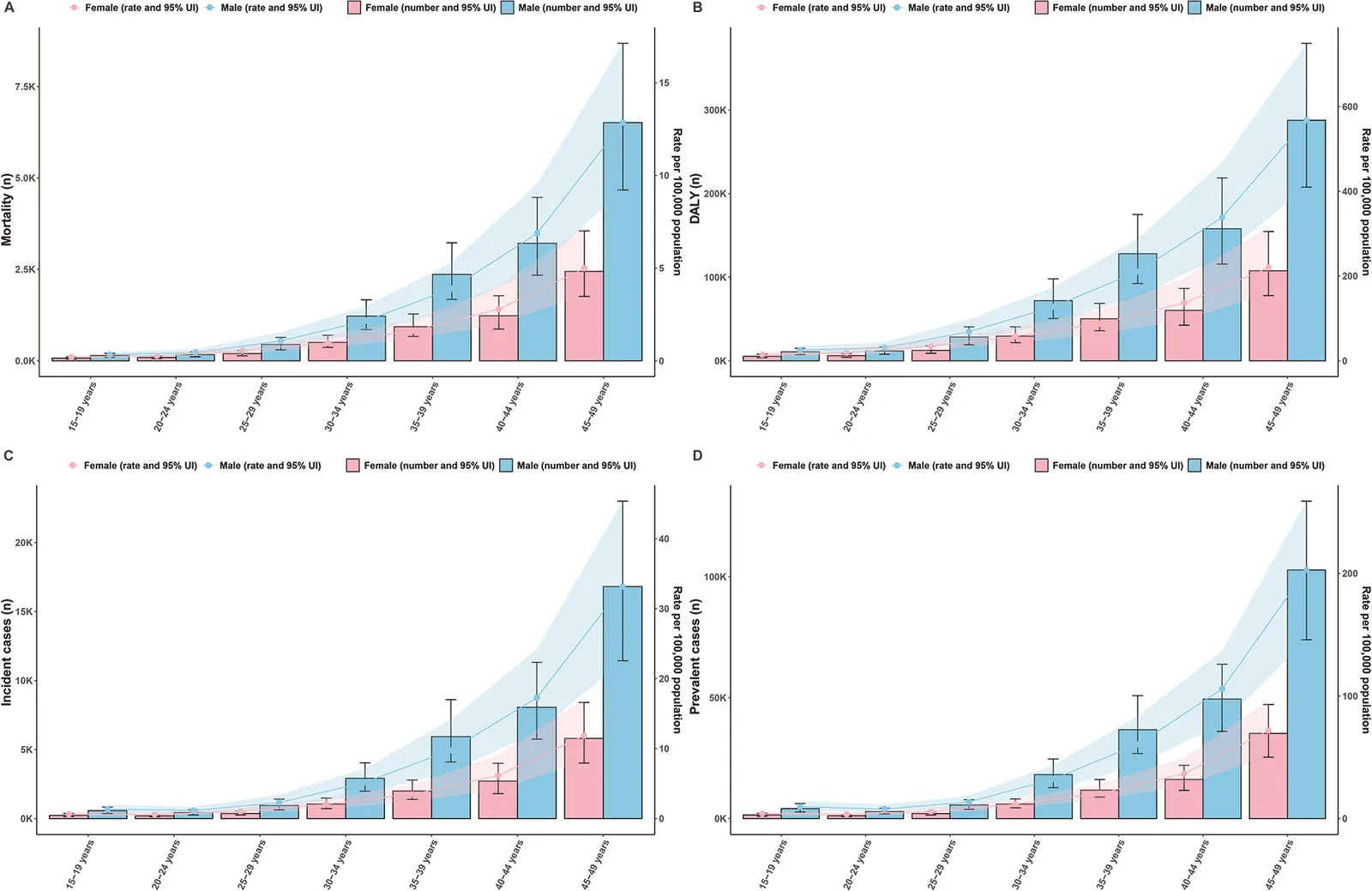
Age- and sex-specific burden of EOCRC in China in 2023, among individuals aged 15–49 years. Panels show **(A)** deaths, **(B)** DALYs, **(C)** incident cases, and **(D)** prevalent cases across seven 5-year age groups (15–19 to 45–49 years). Bars represent absolute case numbers with 95% UIs, while lines with shaded areas denote corresponding ASRs per 100,000 population with 95% UIs. Blue elements represent males, and pink elements represent females, as indicated in the legend. DALY, disability-adjusted life year; UI, uncertainty interval.

For death and DALY burden (**Table 2**; **Supplementary Figures S1A, B**; **Figures 2A, B**), all age subgroups younger than 45 years experienced substantial absolute reductions, and the magnitude of decline weakened gradually with age. The youngest populations (15–19 and 20–24 years) saw the steepest drops in death counts (over 80% reduction), whereas the 45–49 years group was the only subgroup with growing absolute deaths (+27.17%). Similarly, DALY numbers fell sharply in all groups aged 15–44 years, while the 45–49 years subgroup recorded a notable 28.65% rise in DALYs. All age groups had significant downward trends in ASMR and ASDR, with younger age strata showing faster annual declines.

Incidence trends showed stark age divergence (**Table 2**; **Supplementary Figure S1C**; **Figure 2C**). Absolute incident cases declined sharply in populations aged 15–39 years, with the largest reduction (-75.55%) seen in the 20–24 years group. By contrast, the 40–44 and 45–49 years subgroups exhibited rising incident cases, with increases of 30.84% and 114.30%, respectively. ASIR declined significantly in groups younger than 40 years. The 40–44 age group showed a flat trend without statistical significance, whereas the 45–49 age bracket had a statistically significant mild upward trend in ASIR.

Age-specific prevalence patterns were similarly polarized (**Table 2**; **Supplementary Figure S1D**; **Figure 2D**). Absolute prevalent cases declined in all subgroups aged 15–34 years but increased from the 35–39 years group onward, with the maximum growth observed in the 45–49 years group (+180.72%). All 15–34 years subgroups exhibited significant downward ASPR trends, while the 35–39 years group showed a flat non-significant trend (AAPC = 0.01, 95% CI: -0.21 to 0.24). By contrast, the 40–44 and 45–49 years groups had significantly rising ASPRs. The 45–49 years group remained the dominant contributor to total prevalent cases across the entire study period.

In summary, younger EOCRC populations (15–39 years) experienced sustained reductions in absolute burden and falling standardized rates, while the two oldest subgroups (40–44 and 45–49 years) bore expanding incident and prevalent case loads, with the 45–49 years group as the sole age stratum with the fastest rising ASIR and ASPR. EOCRC burden was heavily concentrated in the 45–49 years population, forming a distinct age-dependent gradient of disease burden in China.

### 3.4 Burden attributable to major risk factors

From 1990 to 2023, multiple modifiable dietary and metabolic risk factors were associated with EOCRC burden in China, with divergent temporal patterns of attributable deaths and DALYs (**Table 3**, **Figure 3**). Most risk factors exhibited substantial declines in attributable absolute cases and significant downward age-standardized attributable rates, while high BMI and high processed meat intake saw continuous growth of attributable burden.

**Table 3:** Burden of EOCRC attributable to risk factors in China, 1990-2023.

Measure	Risk factors	Number (1990, 95%UI)	Number (2023, 95%UI)	Percent change (%)	ASR (1990, 95%UI)	ASR (2023, 95%UI)	AAPC of ASR (95% CI)
Deaths	Low milk	4463(1222,7408)	3410(954,5675)	-23.31	0.67(0.18,1.11)	0.53(0.15,0.88)	-0.77 (-1.06 to -0.49)*
	Low whole grains	4191(1661,6487)	3367(1429,5092)	-19.37	0.63(0.25,0.97)	0.52(0.22,0.79)	-0.62 (-0.91 to -0.33)*
	High red meat	3599(0,7643)	3109(0,6136)	NA	0.54(0.00,1.14)	0.48(0.00,0.95)	-0.24 (-0.50 to 0.02)
	High alcohol use	2233(1272,3391)	2041(1087,3091)	-8.28	0.33(0.19,0.51)	0.32(0.17,0.48)	-0.11 (-0.35 to 0.12)
	High body-mass index	1017(462,1734)	1467(731,2394)	44.83	0.15(0.07,0.26)	0.23(0.11,0.37)	1.22 (0.93 to 1.51)*
	Smoking	1362(772,1981)	1345(775,2019)	-0.80	0.20(0.12,0.30)	0.21(0.12,0.31)	0.11 (-0.14 to 0.36)
	High fasting plasma glucose	1718(939,2751)	1213(766,1860)	-29.08	0.26(0.14,0.41)	0.19(0.12,0.29)	-0.76 (-0.97 to -0.55)*
	Low calcium	3325(2057,4691)	1007(681,1417)	-69.56	0.50(0.31,0.70)	0.16(0.11,0.22)	-3.44 (-3.66 to -3.21)*
	High processed meat	250(82,442)	552(186,1095)	120.37	0.04(0.01,0.07)	0.09(0.03,0.17)	2.67 (2.47 to 2.86)*
	Low physical activity	346(169,644)	286(130,556)	-16.94	0.05(0.03,0.10)	0.04(0.02,0.09)	-0.37 (-0.63 to -0.11)*
	Low fiber	539(139,1372)	137(20,399)	-74.43	0.08(0.02,0.21)	0.02(0.00,0.06)	-3.69 (-3.99 to -3.38)*
DALYs	Low milk	228145(62175,379325)	167069(46635,277215)	-26.50	34.12(9.30,56.73)	25.88(7.22,42.94)	-0.70 (-0.94 to -0.46)*
	Low whole grains	213905(84907,329665)	165049(70822,250217)	-22.55	31.99(12.70,49.30)	25.56(10.97,38.76)	-0.64 (-0.90 to -0.38)*
	High red meat	184189(0,391556)	152509(0,303152)	NA	27.55(0.00,58.56)	23.62(0.00,46.95)	-0.51 (-0.77 to -0.24)*
	High alcohol use	115160(66159,176593)	100645(54265,151487)	-12.33	17.22(9.89,26.41)	15.59(8.40,23.46)	-0.32 (-0.55 to -0.10)*
	High body-mass index	52767(24177,90439)	71975(35491,117949)	36.92	7.89(3.62,13.53)	11.15(5.50,18.27)	1.11 (0.83 to 1.39)*
	Smoking	66897(37809,97405)	64488(37030,96640)	-3.21	10.00(5.65,14.57)	9.99(5.74,14.97)	0.12 (-0.12 to 0.36)
	High fasting plasma glucose	85792(46626,138225)	58332(36988,90042)	-31.73	12.83(6.97,20.67)	9.03(5.73,13.95)	-1.01 (-1.25 to -0.77)*
	Low calcium	172041(106397,241525)	49799(33559,69460)	-70.91	25.73(15.91,36.12)	7.71(5.20,10.76)	-3.41 (-3.62 to -3.20)*
	High processed meat	12819(4199,22603)	27121(9097,53056)	111.60	1.92(0.63,3.38)	4.20(1.41,8.22)	2.55 (2.36 to 2.74)*
	Low physical activity	17501(8755,31818)	13891(6579,26670)	-20.38	2.62(1.31,4.76)	2.15(1.02,4.13)	-0.64 (-0.87 to -0.42)*
	Low fiber	27639(7277,69775)	6873(1049,19825)	-75.04	4.13(1.09,10.44)	1.06(0.16,3.07)	-3.89 (-4.18 to -3.59)*

AAPC, average annual percentage change; ASR, age-standardized rate; CI, confidence interval; UI, uncertainty interval; DALY, disability-adjusted life year. NA, not calculated because the 95% UI lower bound was zero. *P < 0.05.

**Figure 3:**
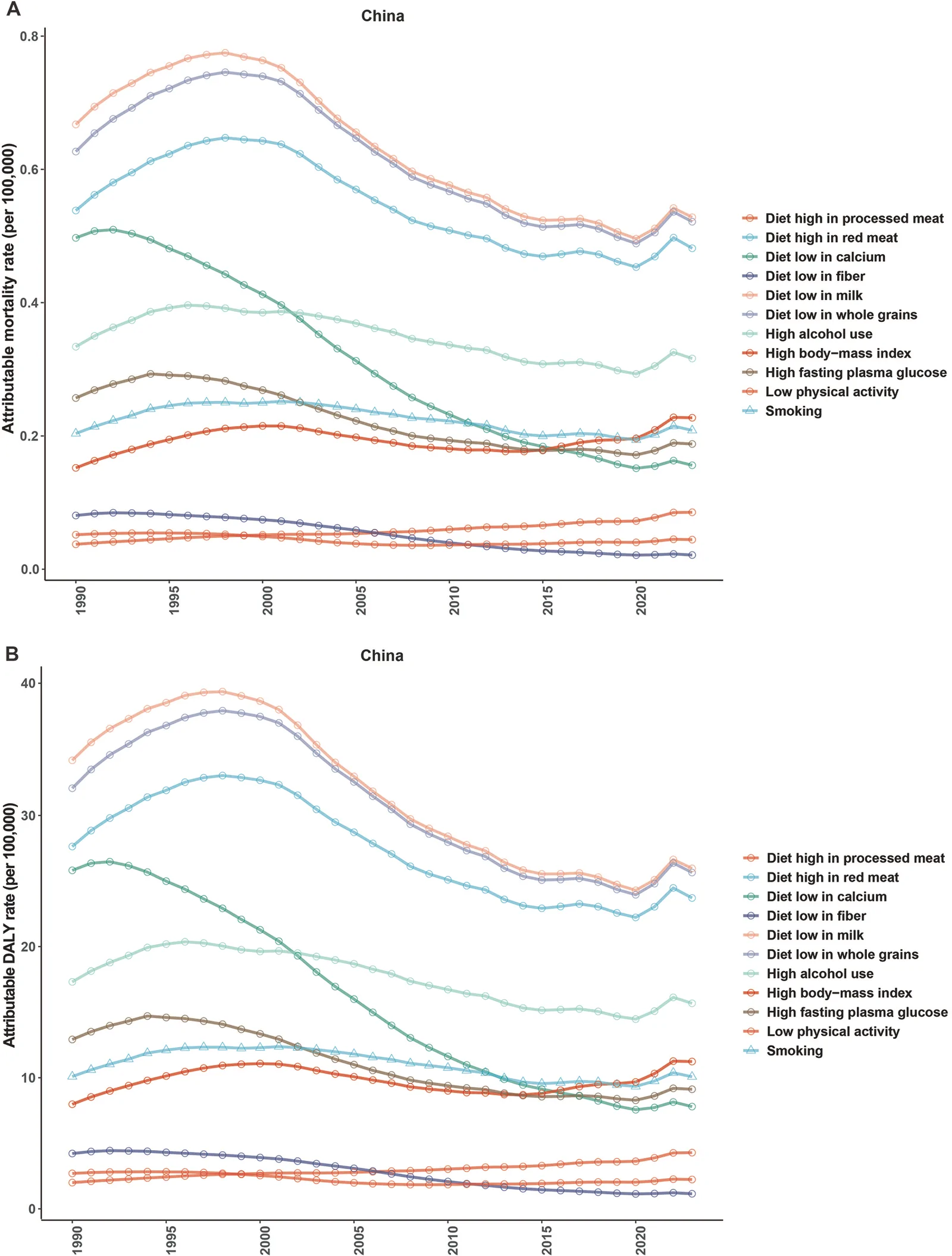
Temporal trends in ASMR and ASDR of EOCRC attributable to modifiable dietary, behavioural and metabolic risk factors in China, 1990-2023. **(A)** shows attributable ASMR, and **(B)** shows attributable ASDR, both expressed per 100,000 population. Each coloured line denotes the disease burden attributable to a single risk factor, with full labels provided in the figure legend. DALY, disability-adjusted life year.

The absolute EOCRC burden attributable to each risk factor varied markedly during the study period (**Table 3**, **Figure 3**). Low fiber intake brought the greatest reduction in attributable deaths and DALYs, falling by 74.43% and 75.04%, respectively, with significantly declining ASMR (AAPC = -3.69) and ASDR (AAPC = -3.89). Low calcium intake showed the second-largest burden reduction, with attributable deaths and DALYs decreasing by 69.56% and 70.91%, accompanied by significant downward trends in relevant ASRs. By contrast, high processed meat intake yielded the sharpest growth in attributable burden: its attributable deaths and DALYs rose by 120.37% and 111.60%, with significantly rising ASMR (AAPC = 2.67) and ASDR (AAPC = 2.55). High BMI also presented notable increases in attributable deaths (+44.83%) and DALYs (+36.92%), with upward AAPCs for standardized rates. High red meat intake, high alcohol use and smoking had non-significant annual changes in ASMR, whereas all other risk factors displayed sustained downward trends. Overall, low milk intake and low whole grains intake remained the top two contributors to EOCRC-attributable mortality and DALY burden.

Consistent age-stratified distributions of attributable ASMR and ASDR were observed across all risk factors. For every risk factor, both attributable rates rose obviously with age and peaked in the 45–49 years group in 1990 (**Supplementary Figures S2A**, **S3A**) and 2023 (**Supplementary Figures S2B**, **S3B**), while the lowest burden was seen in the 15–19 years subgroup. Across all age brackets and two time points, low milk intake and low whole grains consistently generated higher attributable mortality and DALY burden than other exposures, with the largest inter-factor gaps found in the 45-49-year population.

Notably, these results highlight an important difference between risk factor related burden magnitude and temporal trends. Low milk and low whole grains intake represented risk factors with the largest total attributable burden across the study period, whereas high processed meat intake and high BMI were characterised by rapidly growing attributable burden, despite their relatively lower overall burden magnitude.

### 3.5 Temporal trend segments and inflection points (joinpoint regression)

Joinpoint regression analysis (**Table 4**, **Figure 4**) identified non-linear temporal trends with clear inflection points for mortality, DALY, incidence, and prevalence rates of EOCRC between 1990 and 2023.

**Table 4:** Segment-specific APCs for temporal trends in EOCRC rates in China, 1990-2023.

Metric	Year interval	APC (95% CI)	*P*-value
Death	1990-1998	1.41 (1.04, 1.78)	<0.05
	1998-2013	-2.75 (-2.91, -2.59)	<0.05
	2013-2020	-0.70 (-1.23, -0.17)	<0.05
	2020-2023	2.24 (0.22, 4.30)	<0.05
DALY	1990-1998	1.22 (0.87, 1.58)	<0.05
	1998-2013	-3.08 (-3.24, -2.92)	<0.05
	2013-2020	-0.72 (-1.23, -0.20)	<0.05
	2020-2023	2.51 (0.55, 4.51)	<0.05
Incidence	1990-1997	2.58 (2.13, 3.04)	<0.05
	1997-2014	-1.12 (-1.26, -0.98)	<0.05
	2014-2023	2.21 (1.83, 2.59)	<0.05
Prevalence	1990-1997	3.36 (3.02, 3.70)	<0.05
	1997-2015	-0.06 (-0.16, 0.03)	0.20
	2015-2023	2.96 (2.62, 3.30)	<0.05

APC, annual percentage change; CI, confidence interval; DALY, disability-adjusted life year.

**Figure 4:**
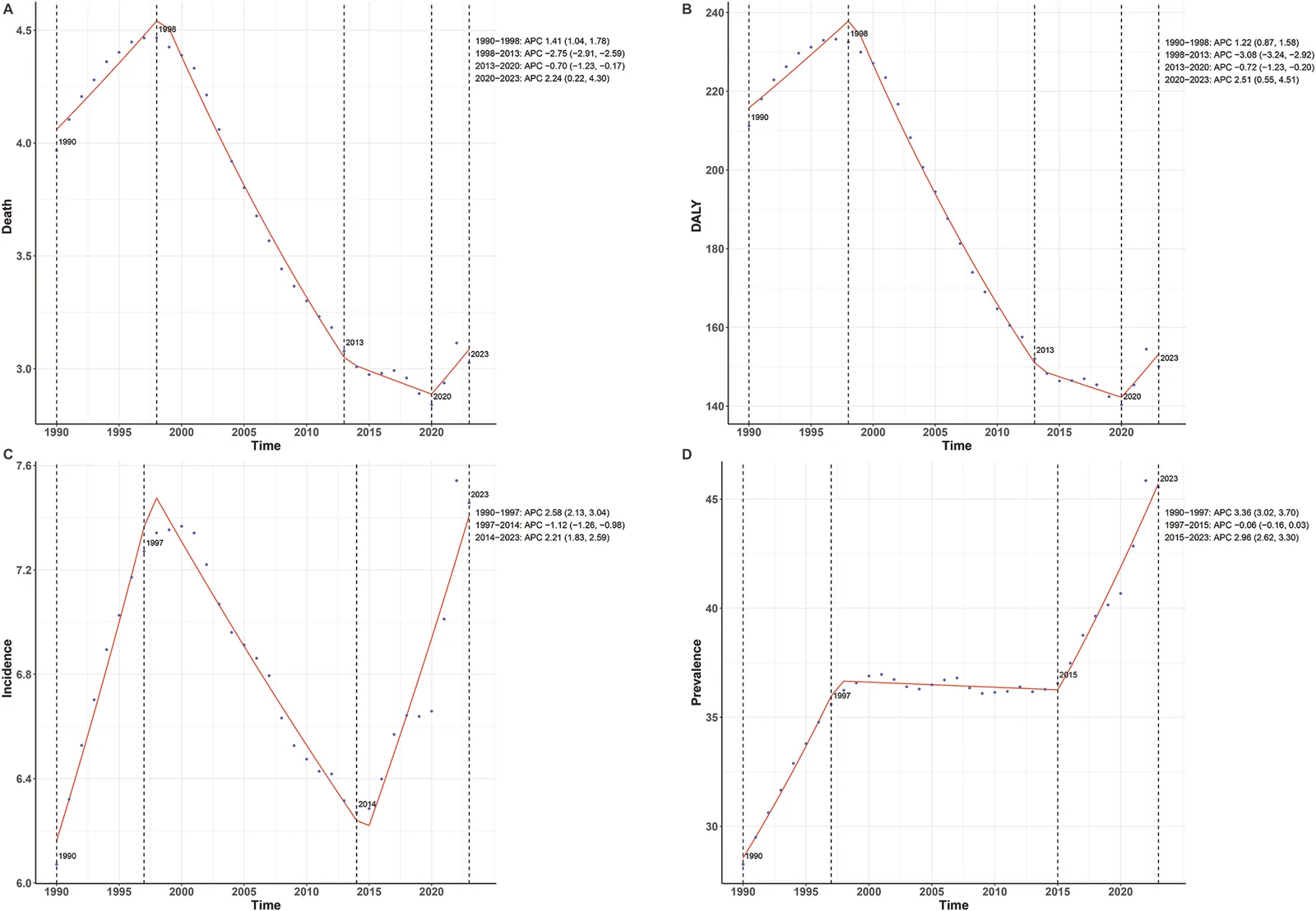
Joinpoint regression analysis of temporal trends in ASRs of EOCRC in China, 1990-2023. Panels present temporal trends in four major EOCRC indicators: **(A)** ASMR, **(B)** ASDR, **(C)** ASIR, and **(D)** ASPR. Blue points indicate annual observed values, red solid lines represent segmented trend fittings, and vertical dashed lines mark statistically significant joinpoints. Segment-specific APCs are shown in each panel and summarized in **Table 4**. APC, annual percentage change; DALY, disability-adjusted life year.

Mortality and DALY rates shared identical segmented structures (**Table 4**, **Figures 4A, B**): both rose from 1990 to 1998 (APC = 1.41 for mortality, APC = 1.22 for DALY), followed by a sharp decline during 1998-2013 (APC = -2.75 and -3.08, respectively), a slower decline from 2013 to 2020 (APC = -0.70 and -0.72), and a subsequent rebound increase from 2020 to 2023 (APC = 2.24 and 2.51).

The incidence rate exhibited three distinct segments: an increase from 1990 to 1997 (APC = 2.58), a sustained decline from 1997 to 2014 (APC = -1.12), and another rising phase from 2014 to 2023 (APC = 2.21) (**Table 4**, **Figure 4C**). For the prevalence rate (**Table 4**, **Figure 4D**), the trend consisted of three segments: continuous growth from 1990 to 1997 (APC = 3.36), a stable flat trend from 1997 to 2015 with non-significant change (APC = -0.06, *P* = 0.20), and a significant rebound increase from 2015 to 2023 (APC = 2.96).

Overall, mortality and DALY rates followed nearly identical segmented trajectories. Incidence and prevalence rates shared similar fluctuation characteristics, featuring an initial rise, a mid-term stable or declining stage, and a prominent rebound in the latest period.

### 3.6 Future projections (2024-2040) based on ARIMA modelling

ARIMA models were constructed using historical data from 1990 to 2023 to project EOCRC mortality and DALY burden trends among Chinese people aged 15–49 years between 2024 and 2040. All projections were presented with 95% PIs (**Figure 5**; **Supplementary Figure S4**).

**Figure 5:**
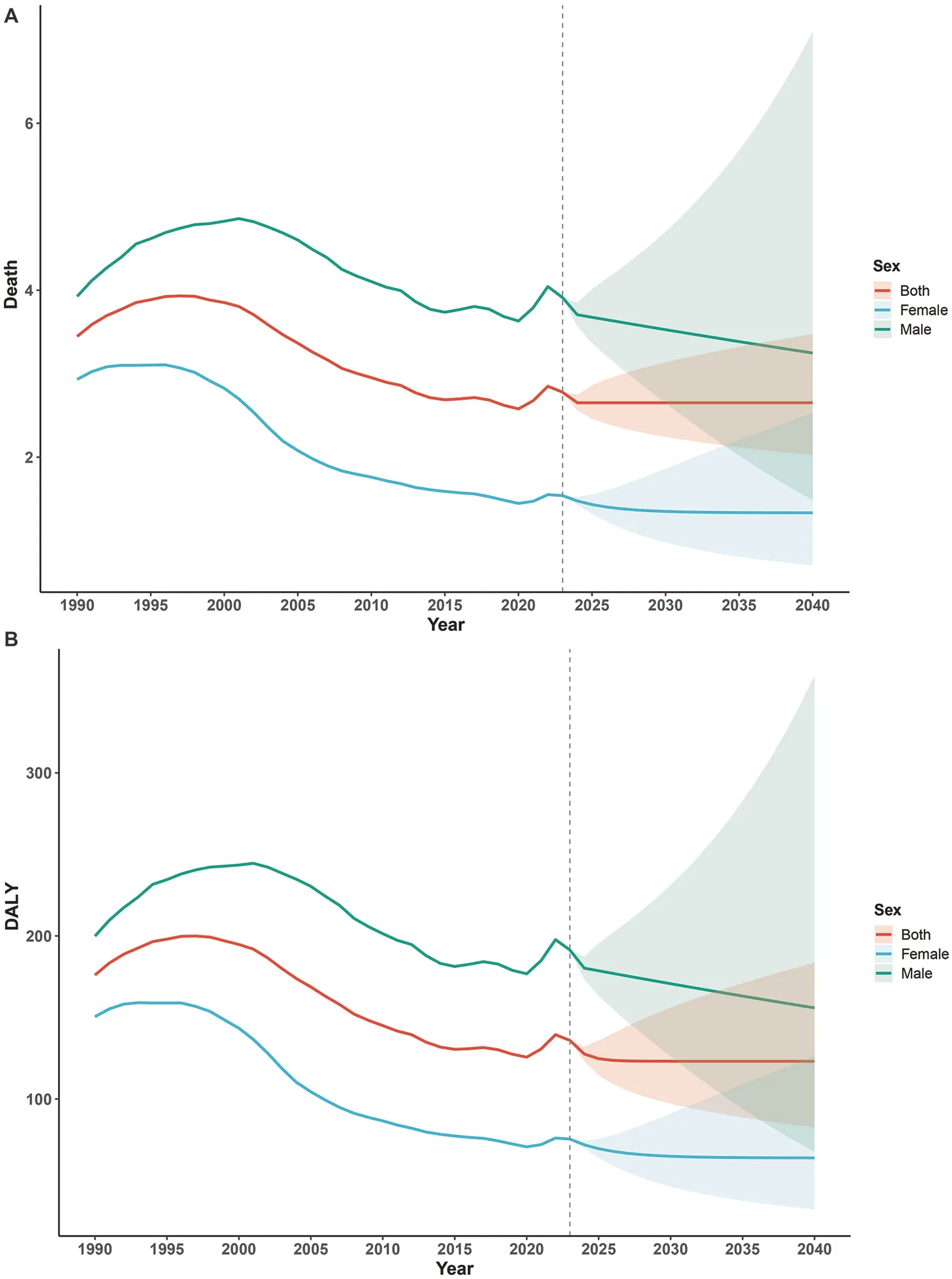
Observed and ARIMA-projected ASRs of EOCRC burden in China, 1990-2040. Panels show **(A)** ASMR and **(B)** ASDR, each presenting observed estimates for 1990–2023 and ARIMA-based projections for 2024-2040. The vertical dashed line marks the transition from observed to projected values. Shaded areas represent 95% PIs for projections. Colors denote sex groups (male, female, and both sexes combined), as indicated in the legend. DALY, disability-adjusted life year; PI, prediction interval.

For ASRs (**Figures 5A, B**), both males and females were predicted to undergo sustained declines in ASMR and ASDR over the forecast period. Males maintained persistently higher ASMR and ASDR compared with females throughout the projection period.

For absolute burden indicators (**Supplementary Figures S4A, B**), overall deaths and DALYs were projected to decrease continuously from 2024 to 2040. Males dominated the absolute burden of EOCRC-related deaths and DALYs. Although absolute burden declined in both sexes, the gender disparity remained evident until 2040.

## 4 Discussion

This study systematically analyzed the epidemiological burden of EOCRC among Chinese residents aged 15–49 years over the 33-year period from 1990 to 2023. We further identified segmented nonlinear inflection points via Joinpoint regression and predicted EOCRC mortality and DALY burden trends up to 2040 using ARIMA models. Overall, national EOCRC exhibited divergent long-term trajectories across four core metrics, remarkable sex and age epidemiological disparities, graded attributable burden associated with multiple modifiable dietary, metabolic, and behavioral exposures, and multi-stage nonlinear temporal shifts; gender gaps in burden will persist through 2040 according to predictive estimations.

From 1990 to 2023, national EOCRC mortality and DALY absolute counts and ASRs decreased continuously, while incident and prevalent cases increased significantly with rising ASRs. The downward trends of mortality and premature death loss align with nationwide improvements in standardized CRC diagnosis, endoscopic screening, and multidisciplinary treatment in recent decades (14). Population-wide CRC screening initiatives and standardized radical therapy could have contributed to the observed decline in population-level EOCRC mortality and the reduction in years lived with disability (10). Nevertheless, the continuous expansion of national EOCRC incident and prevalent case pools, particularly the sharp surge among males, highlights emerging challenges in youth CRC prevention (12). The sustained growth of incidence and prevalence is potentially related to rising metabolic risk exposure among young males, including increasing overweight and obesity and long-term excessive intake of processed meat (33). Unlike mortality benefits gained from clinical progress, rising incidence may reflect elevated baseline carcinogenic exposure among working-age populations rather than improved survival outcomes. The opposite trends across core metrics demonstrate that comprehensive multi-indicator evaluation is essential for fully interpreting the epidemiological landscape of EOCRC in China.

Marked sex disparities existed across all dimensions of EOCRC burden, with completely divergent temporal trajectories between males and females. Males consistently carried higher absolute and standardized mortality, DALY and prevalence burden, achieved smaller burden reductions, and experienced sharp rises in incidence and prevalence, whereas female incidence declined steadily (13). Such prominent gender gaps could be partly attributed to sex-specific differences in modifiable risk exposure. Young Chinese males have higher prevalence of smoking, heavy alcohol consumption, and high-processed-meat dietary patterns, all recognized risk contributors to EOCRC (34). In contrast, females generally maintain lower BMI, higher intake of potentially protective foods such as milk, whole grains and calcium, and lower tobacco and alcohol exposure, which could collectively lower EOCRC risk (11). In addition, women’s higher adherence to routine physical examinations could facilitate early intervention for precancerous lesions, potentially further narrowing mortality and DALY gaps (35). Endogenous hormonal differences also exert protective effects against colorectal carcinogenesis in females and partially counteract external carcinogenic exposures (36). The continuously widening gender gap suggests that gender-stratified targeted prevention centered on working-age males should be prioritized in future national EOCRC control policies.

Clear age-dependent gradient heterogeneity was observed across all 5-year age subgroups aged 15–49 years: EOCRC mortality, DALY, incidence and prevalence risks increased progressively with age, with maximum absolute burden and standardized rates concentrated in the 45–49 years subgroup, while adolescents and young adults aged 15–34 carried relatively mild burden (20). For mortality and DALY, all groups younger than 45 years achieved substantial burden reductions, yet the magnitude of decline weakened with age; only the 45–49 years subgroup presented growing death and DALY cases. This age gradient conforms to the cumulative carcinogen exposure theory: long-term accumulation of unhealthy diet, metabolic disorders and adverse lifestyles gradually elevates cancer risk in middle-aged young adults (7). Incidence and prevalence trends further amplified age heterogeneity: incident and prevalent cases declined significantly in populations aged 15–39 years but rose rapidly in the 40–44 and 45–49 years subgroups, with upward ASIR and ASPR exclusively observed in the two oldest brackets. The exclusive burden expansion in middle-aged young adults suggests that accumulated metabolic and dietary hazards have become important risk contributors for EOCRC in this population (3). Collectively, the stable age gradient suggests that the 45-49-year population may represent a key high-risk group for Chinese EOCRC, warranting further evaluation of targeted screening and health education. However, the present analysis cannot directly establish the effectiveness or optimal age of screening, which remains to be validated in prospective studies.

Multiple modifiable dietary, metabolic and behavioral exposures contributed quantifiable attributable EOCRC burden, with distinct hierarchical contributions and consistent age distribution gradients. Low fiber intake yielded the largest reductions in attributable deaths and DALYs, followed by insufficient calcium and milk intake; high processed meat intake produced the sharpest growth of attributable burden, accompanied by high BMI as another rapidly rising risk factor (37). High red meat consumption, smoking and heavy alcohol use maintained stable burden effects without significant long-term temporal changes in standardized rates. Low milk and low whole grain intake remained the two leading contributors to EOCRC-attributable mortality and DALY throughout the study period, highlighting the potential protective role of balanced dietary patterns in youth CRC prevention (20). Attributable ASMR and ASDR for all risk factors rose steadily with age and peaked in the 45–49 years subgroup, consistent with the overall age gradient of EOCRC burden (20). Sex differences in exposure prevalence to these modifiable factors could represent an important explanation for gender disparities in population-level EOCRC burden (11). These findings provide quantitative epidemiological evidence suggesting that interventions optimizing dietary structure, controlling body weight and restricting processed meat intake hold potential to reduce future EOCRC burden among Chinese young adults.

Joinpoint regression identified distinct nonlinear segmented trends and statistically significant inflection points for all four EOCRC burden metrics. Mortality and DALY rates exhibited identical four-stage trajectories: initial growth (1990-1998), steep decline (1998-2013), moderately slowed decline (2013-2020), and post-pandemic rebound growth (2020-2023). The rising phase before 1998 could be linked to rapid westernization of youth dietary and lifestyle patterns in China, where rising obesity and processed meat consumption potentially contributing to elevated EOCRC risk (8). The sharp decline from 1998 to 2013 is potentially related to the gradual popularization of colonoscopy screening, standardized early diagnosis and radical surgery, and nationwide nutritional health promotion campaigns starting in the late 1990s (14). The slowed decline during 2013–2020 could reflect saturation of short-term preventive gains, whereas the 2020–2023 rebound might be partially associated with COVID-19-related delays in routine physical examination and colorectal lesion screening, which could have postponed early intervention. However, this possibility is drawn from external published literature, and other factors including shifts in population-level lifestyle behaviours and changes in diagnostic practice may also contribute to this upward trend (38). Incidence and prevalence presented three-phase fluctuation patterns: early growth (1990-1997), mid-term decline (1997-2014/2015), and prominent late rebound. The flat prevalence trend from 1997 to 2015 suggests that improved survival offset reductions in new cases and stabilized the national prevalent population scale. The marked rebound in incidence and prevalence after 2014/2015 could reflect resurgent lifestyle-related carcinogenic risks among young populations, potentially eroding previous preventive achievements and calling for sustained long-term CRC prevention targeting young adults (12).

ARIMA predictive results demonstrated that ASMR and ASDR of EOCRC will maintain continuous downward trends for both sexes from 2024 to 2040, with relatively narrow 95% PIs indicating limited forecast uncertainty under the model assumptions. Males will consistently sustain higher ASMR and ASDR than females across the forecast period, and gender burden disparities will persist until 2040. Absolute death and DALY totals are projected to decline steadily over the next 17 years, yet males will continuously dominate the absolute EOCRC burden. Despite the overall favorable downward trend of standardized rates, the persistent male-dominated burden gap indicates that universal one-size-fits-all prevention cannot eliminate gender imbalance; gender-specific interventions targeting young males are indispensable for long-term burden reduction (13).

The present study adopted the latest standardized GBD 2023 population-based dataset to complete a full-spectrum 33-year epidemiological assessment of Chinese EOCRC among residents aged 15–49 years. We comprehensively quantified overall burden trends, systematically explored sex- and five-year age-stratified epidemiological disparities, quantitatively evaluated the attributable burden of multiple dietary, metabolic, and behavioral risk factors, detected nonlinear trend inflection points via Joinpoint regression, and further predicted medium-and-long-term mortality and DALY burden up to 2040 using ARIMA models. The combination of retrospective long-term trend analysis and prospective burden forecasting provides systematic population-level epidemiological evidence for developing hierarchical, precise youth CRC prevention and screening strategies in China.

Several limitations of this research should be acknowledged. First, all GBD estimates are model-derived rather than directly observed, relying on mathematical modelling that integrates multi-source cancer registry data, mortality surveillance, cohort studies, and hospital-based statistics. The quality and completeness of source data may have varied over the 33-year study period, particularly in earlier years and in rural or remote regions with incomplete cancer registration and underreporting, which may introduce bias to national burden estimations. Moreover, changes in cancer registration coverage, diagnostic practices, and disease coding systems over time may affect the temporal comparability of estimates. Risk-attributable estimates are further based on comparative risk assessment assumptions, including the selection of risk-outcome pairs, exposure-response functions, and theoretical minimum risk exposure levels, and represent population-level attributable burden rather than individual-level causal effects. Moreover, this is an ecological analysis based on population-level aggregated data, such that findings cannot be directly extrapolated to individual-level risk or causal relationships (39). Second, this study only analyzed overall EOCRC without further stratification by anatomical subsite, pathological subtype or molecular phenotype; colon and rectal EOCRC may present heterogeneous epidemiological trends and risk factor associations, which were not assessed separately here (5). Third, we only quantified the attributable burden of common modifiable risk factors and did not incorporate non-modifiable contributors including genetic susceptibility, family cancer history and inflammatory bowel disease (3). Fourth, this analysis focused exclusively on national aggregate burden and failed to explore regional, urban-rural or provincial disparities in EOCRC epidemiological characteristics (9, 13). Finally, ARIMA prediction purely extrapolates historical trends under the premise that risk-factor profiles, screening policies and therapeutic performance will remain stable in the future. Large-scale youth-targeted CRC-screening programmes, population-wide dietary-intervention strategies, or therapeutic innovations may substantially alter real-world EOCRC burden trajectories beyond model-based projections.

In conclusion, EOCRC burden among Chinese working-age populations exhibited divergent long-term trends across mortality, DALY, incidence and prevalence metrics from 1990 to 2023. Mortality and premature loss burden declined continuously, whereas incident and prevalent cases expanded significantly, accompanied by persistent prominent sex disparities and a clear age gradient with peak risk in the 45–49 years subgroup. Multiple dietary and metabolic risk factors exerted graded attributable burden, and distinct nonlinear segmented inflection points were identified during the study period. Future projections indicate that standardized mortality and DALY rates will keep declining until 2040, while the male-dominated burden pattern and gender gaps will remain evident. Sustained population-wide primary prevention focusing on modifiable dietary and metabolic exposures, further evaluation of gender- and age-targeted high-risk population screening, and optimized allocation of youth gastrointestinal healthcare resources may help curb rising EOCRC incidence and prevalence and mitigate long-term EOCRC disease burden in China.

## 5 Conclusion

This 33-year national epidemiological analysis of Chinese EOCRC uncovered opposing long-term burden trends, persistent age and gender disparities, and shifting risk-related disease pressures. Though mortality and premature death losses dropped over time, growing incident and prevalent cases, concentrated in males and the 45–49 age group, signal emerging public health threats. Dietary and metabolic risk factors account for most attributable EOCRC burden, and pandemic-related disruptions may have contributed to a recent rebound in disease metrics. Predictions show gender-based burden gaps will persist through 2040 despite falling ASRs. Customized prevention combining further evaluation of age-specific screening, male-oriented lifestyle interventions and population-wide dietary improvements could help address rising EOCRC prevalence and ease long-term disease burden among working-age Chinese residents.

## Data Availability

Publicly available datasets were analyzed in this study. This data can be found here: Data were obtained from the Global Burden of Disease (GBD) 2023 study. The dataset can be accessed at: https://ghdx.healthdata.org/gbd-2023. No formal accession number is assigned to the GBD dataset.

## Supplementary material

Supplementary Figure 1 Age- and sex-specific burden of EOCRC in China in 1990, among individuals aged 15–49 years. Panels show (A) deaths, (B) DALYs, (C) incident cases, and (D) prevalent cases across seven 5-year age groups (15–19 to 45–49 years). Bars represent absolute case numbers with 95% UIs, while lines with shaded areas denote corresponding ASRs per 100,000 population with 95% UIs. Blue elements represent males, and pink elements represent females, as indicated in the legend. DALY, disability-adjusted life year; UI, uncertainty interval.

Supplementary Figure 2 Risk factor-attributable ASMR of EOCRC among the Chinese population in 1990 and 2023. Panel (A): 1990 attributable ASMR; Panel (B): 2023 attributable ASMR. Horizontal bars show attributable ASMR (per 100,000 persons) across 5-year age brackets (15–19 to 45–49 years). Distinct colors denote different modifiable risk factors.

Supplementary Figure 3 Risk-attributable ASDR of EOCRC in China in 1990 and 2023. Panel (A): attributable DALY rates in 1990; Panel (B): attributable DALY rates in 2023. Horizontal bars represent attributable DALY values (per 100,000 persons) across seven 5-year age brackets ranging from 15–19 to 45–49 years. Distinct colors denote different modifiable risk factors.

Supplementary Figure 4 Observed and ARIMA-projected absolute numbers of EOCRC burden in China, 1990-2040. Panels show (A) deaths and (B) DALYs, each presenting observed estimates for 1990–2023 and ARIMA-based projections for 2024-2040. The vertical dashed line marks the transition from observed to projected values. Shaded areas represent 95% PIs for projections. Colors denote sex groups (male, female, and both sexes combined), as indicated in the legend. DALY, disability-adjusted life year; PI, prediction interval.

Supplementary Table 1 ARIMA model specifications and diagnostic test results for EOCRC burden projections, 1990–2023.

Supplementary Table 2 Out-of-sample validation results of ARIMA models, 1990–2023.
