# Supplementary material for: Early-onset colorectal cancer burden in China from 1990 to 2023: a systematic analysis based on the Global Burden of Disease Study 2023
Source: Front Oncol. 2026 Oct 12;16:1958227. doi: 10.3389/fonc.2026.1958227 (PMC13696216; doi:10.3389/fonc.2026.1958227)
Supplement: Supplementary file 1 [file Table1.docx]

**Supplementary Table 1**. ARIMA model specifications and diagnostic test results for EOCRC burden projections, 1990-2023

| **Metric** | **Indicator** | **Sex** | **ARIMA (p,d,q)** | **AIC** | **KPSS statistic** | **KPSS**  ***P*-value** | **Ljung-Box**  ***P*-value** |
| --- | --- | --- | --- | --- | --- | --- | --- |
| Rate | ASMR | Both | (0,1,1) | -166.948 | 0.827 | 0.010 | 0.746 |
|  | ASMR | Male | (0,2,2) | -155.903 | 0.600 | 0.023 | 0.646 |
|  | ASMR | Female | (1,1,1) | -173.231 | 0.894 | 0.010 | 0.843 |
|  | ASDR | Both | (1,1,1) | -166.403 | 0.837 | 0.010 | 0.900 |
|  | ASDR | Male | (0,2,2) | -155.462 | 0.660 | 0.017 | 0.713 |
|  | ASDR | Female | (1,1,1) | -172.823 | 0.895 | 0.010 | 0.865 |
| Number | Deaths | Both | (0,2,0) | -154.963 | 0.781 | 0.010 | 0.333 |
|  | Deaths | Male | (0,2,2) | -154.369 | 0.504 | 0.041 | 0.529 |
|  | Deaths | Female | (0,2,0) | -166.873 | 0.905 | 0.010 | 0.934 |
|  | DALYs | Both | (0,2,0) | -154.627 | 0.805 | 0.010 | 0.384 |
|  | DALYs | Male | (0,2,0) | -148.583 | 0.575 | 0.025 | 0.284 |
|  | DALYs | Female | (0,2,0) | -166.320 | 0.908 | 0.010 | 0.933 |

Abbreviations: AIC, Akaike information criterion; KPSS, Kwiatkowski-Phillips-Schmidt-Shin; ASMR, age-standardized mortality rate; ASDR, age-standardized DALY rate. KPSS *P* < 0.05 indicates non-stationarity of the original series, which was resolved by differencing; Ljung-Box *P* > 0.05 indicates that residuals were white noise.
