# Supplementary material for: Early-onset colorectal cancer burden in China from 1990 to 2023: a systematic analysis based on the Global Burden of Disease Study 2023
Source: Front Oncol. 2026 Oct 12;16:1958227. doi: 10.3389/fonc.2026.1958227 (PMC13696216; doi:10.3389/fonc.2026.1958227)
Supplement: Supplementary file 2 [file Table2.docx]

**Supplementary Table 2.** Out-of-sample validation results of ARIMA models, 1990-2023

| **Metric** | **Indicator** | **Sex** | **ARIMA (p,d,q)** | **RMSE** | **MAE** | **MAPE** |
| --- | --- | --- | --- | --- | --- | --- |
| Rate | ASMR | Both | (0,1,1) | 0.103 | 0.086 | **3.13%** |
|  | ASMR | Male | (0,2,2) | 0.211 | 0.175 | **4.48%** |
|  | ASMR | Female | (1,1,1) | 0.111 | 0.081 | **5.26%** |
|  | ASDR | Both | (1,1,1) | 8.960 | 6.974 | **5.14%** |
|  | ASDR | Male | (0,2,2) | 11.636 | 9.380 | **4.90%** |
|  | ASDR | Female | (1,1,1) | 5.689 | 4.111 | **5.47%** |
| Number | Deaths | Both | (0,2,0) | 1357.190 | 1059.560 | **5.83%** |
|  | Deaths | Male | (0,2,2) | 868.774 | 704.246 | **5.27%** |
|  | Deaths | Female | (0,2,0) | 439.000 | 318.131 | **6.65%** |
|  | DALYs | Both | (0,2,0) | 69447.604 | 53475.519 | **6.02%** |
|  | DALYs | Male | (0,2,0) | 48197.464 | 37913.871 | **5.80%** |
|  | DALYs | Female | (0,2,0) | 21508.157 | 15561.648 | **6.64%** |

Abbreviations: RMSE, root mean square error; MAE, mean absolute error; MAPE, mean absolute percentage error; ASMR, age-standardized mortality rate; ASDR, age-standardized DALY rate.
